# Payments From Pharmaceutical Companies to Authors Involved in the Valsartan Scandal in Japan

**DOI:** 10.1001/jamanetworkopen.2019.3817

**Published:** 2019-05-17

**Authors:** Toyoaki Sawano, Akihiko Ozaki, Hiroaki Saito, Yuki Shimada, Tetsuya Tanimoto

**Affiliations:** 1 Department of Surgery, Minamisoma Municipal General Hospital, Fukushima, Japan; 2 Department of Breast Surgery, Jyoban Hospital of Tokiwa Foundation, Iwaki, Fukushima, Japan; 3 Medical Governance Research Institute, Shinagawa, Tokyo, Japan; 4 Department of Gastroenterology, Sendai Kousei Hospital, Sendai, Miyagi, Japan; 5 Department of Neurosurgery, Minamisoma Municipal General Hospital, Fukushima, Japan

## Abstract

**Importance:**

Financial relationships between pharmaceutical companies and physicians can bias the conduct, findings, and reporting of clinical trials. In Japan, the valsartan scandal was a significant event: all 5 key articles on the valsartan clinical trials were retracted after the revelation of data falsification. Consequently, the Japan Pharmaceutical Manufacturers Association issued transparency guidelines, and pharmaceutical payments have been publicly disclosed since 2013. However, the distribution of payments from pharmaceutical companies among authors involved in the valsartan scandal after its revelation has not been studied to date.

**Objective:**

To identify the characteristics and distribution of payments from pharmaceutical companies to researchers involved in the valsartan scandal in Japan by using a comprehensive payment database.

**Design, Setting, and Participants:**

This retrospective cross-sectional study, conducted from January 1, 2016, through September 30, 2017, used information on payments from 78 pharmaceutical companies to 50 authors of 5 articles retracted after the valsartan scandal: the Kyoto Heart Study, Jikei Heart Study, SMART (Shiga Microalbuminuria Reduction Trial), VART (Valsartan Amlodipine Randomized Trial), and Nagoya Heart Study. Payments from companies to physicians were analyzed during the period from January 1, 2016, to September 30, 2017.

**Main Outcomes and Measures:**

The main outcomes were the amount and proportion of payments made by pharmaceutical companies to authors, and the forms of these payments.

**Results:**

Of 50 eligible authors, 30 (60%) received payments from pharmaceutical companies in 2016. The payments in the Japanese yen totaled ¥67.15 million (US $590 896) and the mean (SD) payment was more than ¥1.34 million (¥3.01 million) (US $11 817 [$27 292]). Five authors (10%) received more than ¥5 million (US $44 000) and 3 authors (6%) received more than ¥10 million (US $88 000). The total payments to the corresponding author of each article was ¥29 million (US $255 307), accounting for 43.2% of the total payments. Regarding forms of the payments, lecture fees accounted for 81.3% of the total payment (¥54.56 million).

**Conclusions and Relevance:**

Many authors involved in the valsartan scandal received payments from the pharmaceutical industry. Although whether such payments to these authors have decreased since the scandal is unclear, the findings appear to demonstrate that their financial relationships with the industry are still prominent. The findings of the study appear to support the need to encourage more transparency in such relationships because misconduct can occur within unclear relationships.

## Key Points

**Question:**

What is the extent of payments from pharmaceutical companies to authors who wrote the 5 retracted articles on the valsartan clinical trials after the revelation of the scandal in 2013?

**Findings:**

A retrospective cross-sectional analysis using pharmaceutical companies’ data in 2016 showed that 30 of 50 authors (60%) associated with the 5 articles received payments from pharmaceutical companies, with 10% of authors receiving payment in the Japanese yen of more than ¥5 million. The total payment to the corresponding author of these 5 articles accounted for 43.2% of the total payments.

**Meaning:**

Study finding suggest that clinical trial practitioners received large payments from the pharmaceutical industry even after the unprecedented scandal.

## Teaser

This cross-sectional analysis examines the characteristics and distribution of payments from pharmaceutical companies to researchers involved in the valsartan scandal in Japan.

## Introduction

Financial relationships between pharmaceutical companies and physicians can bias the conduct, findings, and reporting of clinical trials.^1^ This problem is well illustrated in the misconduct in Japanese clinical trials of valsartan (hereafter, the valsartan scandal), an angiotensin receptor blocker manufactured by Novartis.^2^ With financial and personnel support from Novartis, 5 Japanese medical schools conducted the clinical trials of valsartan in the 2000s, namely, the Kyoto Heart Study (Kyoto Prefectural Medical University),^3^ Jikei Heart Study (Jikei University of Medicine),^4^ Shiga Microalbuminuria Reduction Trial (SMART) (Shiga University of Medical Science),^5^ Valsartan Amlodipine Randomized Trial (VART) (Chiba University),^6^ and Nagoya Heart Study (Nagoya University)^7^ (Table 1). Their findings were published in international medical journals: the Jikei Heart Study was published in *The Lancet* in 2007,^4^ and the Kyoto Heart Study was published in *European Heart Journal* in 2009.^3^ However, these 5 primary and 3 associated studies, including a subanalysis of the Kyoto Heart Study, were retracted after the revelation of the originally unmentioned involvement of a Novartis employee (statistician) in these studies, and numerous instances of data fabrication.

**Table 1.** Details of the 5 Retracted Articles in the Valsartan Scandal

Clinical Trial	Study Period	Published Journal	Published Date	Retracted Date
Kyoto Heart Study^3^	January 2004 to June 2007	*European Heart Journal*	August 31, 2009	April 7, 2013
Jikei Heart Study^4^	January 2002 to December 2004	*The Lancet*	April 26, 2007	September 6, 2013
SMART^5^	December 2003 to March 2006	*Diabetes Care*	March 15, 2007	March 1, 2014
VART^6^	July 2002 to February 2006	*Hypertension Research*	October 7, 2010	November 3, 2016
Nagoya Heart Study^7^	October 2004 to January 2009	*Hypertension*	January 9, 2012	August 8, 2018

Abbreviations: SMART, Shiga Microalbuminuria Reduction Trial; VART, Valsartan Amlodipine Randomized Trial.

The domestic effect of the valsartan scandal was significant. The Ministry of Health, Labour and Welfare criminally charged Novartis and its employee for using fabricated results of the Kyoto Heart Study and Jikei Heart Study in their advertisement for valsartan in November 2014. They were later acquitted on charges of fraudulent and exaggerated advertising. Japan’s new Clinical Research Act, enforced in April 2018, legally defines the term *clinical research* as clinical trials designed to establish the efficacy of drugs or medical devices. However, this new legal definition is not consistent with the global consensus on clinical research, which covers a wider range of studies, including observational studies. As of March 2019, the case against Novartis is being heard by the Japanese Supreme Court. A series of illegal activities was also covered in international media outlets, including the *Wall Street Journal* and *The New York Times*. Consequently, a new law was enforced in April 2018 to regulate clinical research.

In 2011, the Japan Pharmaceutical Manufacturers Association (JPMA) published transparency guidelines requiring disclosure of all payments to authors for lectures, writing, and consulting, to be published with individuals’ names and affiliations.^8^ Thus, the 71 companies belonging to the JPMA voluntarily began publishing their data on payments to authors since 2013. However, JPMA’s platform for such disclosure is not user friendly and has been inconsistent from company to company. For example, most companies publish their data in the form of images, which makes it difficult to aggregate the data. Moreover, to use a company’s data, an individual must be registered on the company’s website. Such conditions hampered analysis of payments to individual physicians. Although the valsartan scandal should have made the financial relationships between pharmaceutical companies and physicians more subtle, overall, they remained prolific; total payments by the 10 companies with the highest total disbursements to physicians have decreased only slightly, from the Japanese yen of ¥12.9 billion (US $113 million) in 2012 to ¥11.4 billion (US $100 million) in 2016.^9^ It is thus important to clarify the financial relationships among relevant stakeholders after the valsartan scandal, including the individuals and institutions involved in the valsartan scandal. However, to date, little research has been conducted in this area. The aim of this study is to identify the characteristics and distribution of pharmaceutical companies’ payments to valsartan scandal researchers after the scandal, using a comprehensive payment database for Japan constructed by our team for fiscal year 2016.

## Methods

### Study Design, Participants, and Setting

The participants in our study were the authors of the aforementioned 5 key articles that were retracted after the valsartan scandal: the Kyoto Heart Study,^3^ the Jikei Heart Study,^4^ SMART,^5^ VART,^6^ and the Nagoya Heart Study^7^ (Table 1). Valsartan was manufactured by Novartis and approved in the Japanese national health insurance system in November 2000. In 2012, annual domestic sales of valsartan peaked, reaching ¥140 billion (US $1232 million). This study was approved retrospectively by the Institutional Review Board of the Medical Governance Research Institute. The Institutional Review Board waived the need for informed consent from study participants because all the descriptive data collected were publicly available. We followed the Strengthening the Reporting of Observational Studies in Epidemiology (STROBE) reporting guideline.

### Sources of Payment Data

We collected payment data from the disclosures of all 78 companies. These companies are categorized into 71 active JPMA members, 6 subsidiaries of these companies, and 1 past member. For most of these companies, the most recent data are from 2016; moreover, each company updates its payment data and deletes old data annually. The companies included in this study, as well as the starting and ending dates of their payment data, are listed in eTable in the Supplement.

We obtained each company’s payment data and generated a unified single database from them, as follows. First, because no data were published in the form of a spreadsheet, data with character codes were converted into a spreadsheet format. Second, data with no character code were converted into text files using an optical character reader. Third, for data protected against facsimile or reproduction, we used FullShot, version 10 software (Inbit Inc) to scan the data and convert the resultant images into text files. Finally, we confirmed that the transformed data were accurately converted by comparing them with the original data. Our database included physicians’ names, their main institutions, payments received, the form of payments, and the total amount of payments. The form of payment was categorized into the following 4 types: payment for lecture, payment for writing, consulting fees, and others. The data did not include research payments, honoraria, and meal benefits.

### Data Collection

We identified payments from pharmaceutical companies for all the observed authors and formulated descriptive analyses of the characteristics and values of these payments. We further extracted the names of all individuals who received payments of ¥0.5 million, ¥5 million, ¥10 million, or more from any pharmaceutical company. The cutoff values of ¥0.5 million and ¥5 million are used by the Pharmaceutical Affairs and Food Sanitation Council of the Ministry of Health, Labour and Welfare to determine whether committee members should be granted voting rights; if a member receives a payment of ¥0.5 million or more and less than ¥5 million from a single pharmaceutical company, he or she is not granted voting rights in the committee; if a committee member receives a payment of ¥5 million or more from a single pharmaceutical company, he or she cannot be a committee member at all.

## Results

Analyzing the data for all 50 authors of the 5 studies (Table 2), we found that the payments totaled ¥67 147 277 million (US $590 896) and the mean (SD) payment was more than ¥1 342 946 (¥3.10 million) (US $11 817 [$27 292]). Thirty authors (60%) received at least 1 payment in 2016, 5 authors (10%) received more than ¥5 million (US $44 000), and 3 authors (6%) received more than ¥10 million (US $88 000). The total payment to the corresponding authors of each article was ¥29 012 254 (US $255 307), accounting for 43.2% of the total payments to all 50 authors. However, 2 of these 5 corresponding authors (40%) had already resigned from their post to take responsibility for the valsartan scandal, and, thus, hardly received any of the payments. Only 1 author received a payment from Novartis (¥111 370 [US $980]). According to the information disclosed by Novartis, the total value of the company’s payments to researchers decreased from ¥1558 million (US $13.7 million) in 2012 to ¥787 million (US $6.9 million) in 2016.^10^

**Table 2.** Summary of Pharmaceutical Payment to Authors of Valsartan-Related Articles^a^

Characteristic	Kyoto Heart Study	Jikei Heart Study	SMART	VART	Nagoya Heart Study	Total
No. of authors	9	18	4	7	12	50
Authors receiving payment, No. (%)						
≥1 Payment	6 (67)	12 (67)	3 (75)	2 (29)	7 (58)	30 (60)
≥¥500 000	1 (11)	6 (33)	3 (75)	1 (14)	4 (33)	15 (30)
≥¥5 000 000	0	2 (11)	1 (25)	1 (14)	1 (8)	5 (10)
≥¥10 000 000	0	0	1 (25)	1 (14)	1 (8)	3 (6)
Total payment from pharmaceutical companies						
Japanese, ¥	2 051 195	18 743 239	16 619 227	11 288 019	18 445 597	67 147 277
United States, $	18 051	164 941	149 249	99 335	162 321	590 896
Mean (SD) payment from pharmaceutical companies						
Japanese, ¥	227 910.6 (457 280.3)	1 041 291 (1 736 665)	4 154 807 (4 997 619)	1 612 574 (4 241 966)	1 676 872 (4 329 916)	1 342 946 (3 101 395)
United States, $	2005.6 (4024.1)	9163.4 (15 282.7)	36 562.3 (43 979.0)	14 190.7 (37 329.3)	14 756.5 (38 103.3)	11 817.9 (27 292.3)
Median (range) payment to individual authors						
Japanese, ¥	60 000 (0-1 427 736)	194 329 (0-5 386 921)	2 648 588 (0-11 322 051)	0 (0-11 232 334)	76 353.5 (0-14 641 516)	109 664.50 (0-14 641 516)
United States, $	528 (0-12 564.1)	1710.1 (0-47 404.9)	23 308.6 (0-99 634)	0 (0-98 844.5)	771.9 (0-128 845.3)	965.0 (0-128 845.3)
Payment to corresponding authors						
Japanese, ¥	1 427 736	0	1 710 668	11 232 334	14 641 516	29 012 254
United States, $	12 564.1	0	15 053.9	98 844.5	128 845.3	255 307.8
Total payment from Novartis to authors						
Japanese, ¥	0	111 370	0	0	0	111 370
United States, $	0	980	0	0	0	980

Abbreviations: SMART, Shiga Microalbuminuria Reduction Trial; VART, Valsartan Amlodipine Randomized Trial.

^a^ Currency exchange rate is $0.0088 per ¥1 (as of November 6, 2018).

Table 3 shows the payments to the authors by form or payment. Lecture fees accounted for as high as 81.3% of the total payments (¥54 564 808 [US $480 170]) to all 50 authors. The next highest amounts of payments were in the form of consulting fees, at 11.8% (¥7.89 million [US $69 444]), and writing fees, at 5.7% (¥3.86 million [US $33 931]).

**Table 3.** Form of Pharmaceutical Payment to Authors of Valsartan-Related Articles^a^

Characteristic	Kyoto Heart Study	Jikei Heart Study	SMART	VART	Nagoya Heart Study	Total
No. of authors	9	18	4	7	12	50
Authors receiving any payment, No. (%)	6 (67)	12 (67)	3 (75)	2 (29)	7 (58)	30 (60)
Fee, No. (%)						
Speaking	6 (66)	12 (67)	3 (75)	2 (29)	7 (58)	30 (60)
Writing	0	5 (28)	2 (50)	1 (14)	2 (17)	10 (20)
Consulting	1 (11)	4 (22)	3 (75)	1 (14)	2 (17)	11 (22)
Sum of payment						
Speaking fee						
Japanese, ¥	1 973 295	15 766 228	12 941 100	8 311 968	15 572 217	54 564 808
United States, $	17 365	138 743	113 882	73 145	137 036	480 170
Writing fee						
Japanese, ¥	0	1 845 370	1 090 964	301 393	618 076	3 855 803
United States, $	0	16 239	9600	2652	5439	33 931
Consulting fee						
Japanese, ¥	77 900	797 531	2 364 423	2 674 658	1 876 924	7 891 390
United States, $	686	7018	20 807	23 537	16 517	69 444
Unknown						
Japanese, ¥	0	334 110	222 740	0	278 426	835 276
United States, $	0	2940	1960	0	2450	7350
Total payment from pharmaceutical companies						
Japanese, ¥	2 051 195	18 743 239	16 619 227	11 288 019	18 445 597	67 147 277
United States, $	18 051	164 941	149 249	99 335	162 321	590 896

Abbreviations: SMART, Shiga Microalbuminuria Reduction Trial; VART, Valsartan Amlodipine Randomized Trial.

^a^ Currency exchange rate is $0.0088 per ¥1 (as of November 6, 2018).

## Discussion

We found that 60% of the physicians involved in the valsartan scandal received at least 1 pharmaceutical payment in 2016, with a mean payment value exceeding ¥1.34 million (US $11 817). The corresponding authors of the 5 key articles received a total of ¥29 million (US $255 307), accounting for 43.2% of the total payments in 2016, while 2 of the 5 authors (40%) had already resigned their post to take responsibility for the scandal and hardly received any of the payments.

This study has data limitations; thus, whether pharmaceutical payments to authors have changed since the valsartan scandal is unclear. However, this study clearly demonstrates that authors’ financial relationships with the pharmaceutical industry are still prominent. Only 1 author received payment from Novartis; therefore, it may be reasonable to assume that these authors had multiple payment sources other than Novartis. For example, 2 corresponding authors received more than ¥10 million in payments, and are currently professors at Nagoya University and University of Tokyo; the corresponding authors of the VART study (Chiba University) moved to the University of Tokyo in 2012. Both schools have been recognized as top medical schools in Japan, with notable traditions and contributions to the Japanese medical society. We speculate that the title of professor at these universities could be a lucrative facilitator through which companies can promote their products to general physicians; in turn, the professors also enjoy benefits of the roles provided by the companies, which enhance their reputation and income.

According to the information disclosed by Novartis, the total value of the company’s payments to researchers decreased from ¥1558 million (US $13.7 million) in 2012 to ¥787 million (US $6.9 million) in 2016^10^; despite this reduction, the amount of payments could still be regarded as significant. This finding would indicate that Novartis has continuously provided payments to physicians and medical specialists other than those related to the valsartan scandal. As the company with the third largest pharmaceutical sales in the world,^11^ Novartis produces a diverse array of drugs, including agents for diabetes and anticancer drugs. Thus, even after the valsartan failure, which was significant, Novartis can still afford to make large payments to physicians in specialties other than hypertension.

Given such deep-rooted financial ties between pharmaceutical companies and researchers, the enforcement of the Clinical Trials Acts (*rinsho-kenkyu-hou*) in 2018 was unavoidable.^12^ Under the Clinical Trials Acts, both pharmaceutical companies and researchers are required to disclose financial relationships during clinical trials, and punishment has been introduced to regulate potentially similar crimes. The intention of this act in Japan, and of the adoption of Open Payments in the United States, is to ensure greater transparency and/or reduce payments that might otherwise facilitate research misconduct. However, although Open Payments has successfully encouraged transparency, pharmaceutical payments to researchers have not necessarily been reduced.^13,14,15^ Similarly, in Japan, open platforms such as Open Payments, which provide details of physicians’ financial relationships with the pharmaceutical industry, could promote transparency of such relationships; nevertheless, how to maintain fair relationships between researchers and pharmaceutical companies remains to be discussed. There is an urgent need to broaden this discussion in Japan.

Despite widespread criticism about conflicts of interest after the valsartan scandal, financial relationships between physicians and the pharmaceutical industry are still common and opaque. The existence of financial relationships between physicians and the pharmaceutical industry is not necessarily unfavorable by itself; however, it is important to encourage transparency in such relationships, and to accurately disclose payments as much as possible, because undisclosed relationships may potentially promote misconduct.

### Limitations

To our knowledge, this study is the first investigation of pharmaceutical payments for authors involved in the valsartan scandal in Japan. However, it has some limitations. First, this study focuses on payments only in 2016. Although we are interested in payments in previous years, because the authors may have received substantial payments before the valsartan scandal, there are no data accessible data before 2016, because each company updates its payment data and deletes old data annually. We used data from 2016, as these are the most recent data. Second, we collected payment data as published by all 71 companies of the JPMA; however, related data from non-JPMA pharmaceutical companies were not included.

## Conclusions

Of the 50 authors involved in the valsartan scandal, 30 (60%) received pharmaceutical payments 4 years after the scandal was revealed. We believe that a more sophisticated disclosure of pharmaceutical payments is needed. Efforts on these lines may help reduce research misconduct in the future.

## Notes

Supplement. **eTable.** 78 Pharmaceutical Companies and Their Period of Aggregating the Payments
