# Error in Payments From Some Pharmaceutical Companies

**DOI:** 10.1001/jamanetworkopen.2019.8619

**Published:** 2019-07-19

In the Original Investigation titled “Payments From Pharmaceutical Companies to Authors Involved in the Valsartan Scandal in Japan,”^1^ published May 17, 2019, payments from some pharmaceutical companies were double-counted. A complete itemization of corrections is as follows: (1) the number of authors who received payments and the percentage of payments accounted for by corresponding authors in the Key Points; (2) the number of authors who received payments, the percentage of payments accounted for by corresponding authors, and the payment value in the abstract; (3) the number of authors who received payments, the percentage of payments accounted for by corresponding authors, and the payment value in the Results section; (4) the percentage of authors who received payments, the percentage of payments accounted for by corresponding authors, and the payment value in the Discussion section; (5) the number of authors who received payments in the Conclusions section; (6) the payment value and the number of authors who received payments in Table 2; and (7) the payment value and the number of authors who received payments in Table 3. This article has been corrected.^1^
