# Supplementary material for: Payments From Pharmaceutical Companies to Authors Involved in the Valsartan Scandal in Japan
Source: JAMA Netw Open. 2019 May 17;2(5):e193817. doi: 10.1001/jamanetworkopen.2019.3817 (PMC6537813; doi:10.1001/jamanetworkopen.2019.3817)
Supplement: Supplement. — eTable. 78 Pharmaceutical Companies and Their Period of Aggregating the Payments [file jamanetwopen-2-e193817-s001.pdf]

Supplementary Online Content

Sawano T, Ozaki A, Saito H, Shimada Y, Tanimoto T. Payments from pharmaceutical companies to authors involved in the valsartan scandal in Japan. *JAMA Netw Open.* 2019;2(5):e193817. doi:10.1001/jamanetworkopen.2019.3817

eTable. 78 Pharmaceutical Companies and Their Period of Aggregating the Payments

This supplementary material has been provided by the authors to give readers additional information about their work.

© 2019 Sawano T et al. *JAMA Network Open.*

eTable. 78 Pharmaceutical Companies and Their Period of Aggregating the Payments

Pharmaceutical company	Period of the payment data in 2016	
	Starting date	Ending date
Maruho Co., Ltd.,	October 1, 2016	September 30, 2017
Shire Japan KK,	January 1, 2016	December 31, 2016
Fuso Pharmaceutical Industries, Ltd.,	April 1, 2016	March 31, 2017
POLA-Pharma.,	January 1, 2016	December 31, 2016
Nippon Zoki Pharmaceutical Co., Ltd.,	April 1, 2016	March 31, 2017
Nippon Kayaku Co., Ltd.	April 1, 2016	March 31, 2017
Kowa Company. Ltd.,	April 1, 2016	March 31, 2017
Kracle Holdings, Ltd.,	January 1, 2016	December 31, 2016
Fujimoto Pharmaceutical Corporation,	July 1, 2016	June 30, 2017
Kyoto Pharmaceutical Industries, Ltd.	June 1, 2016	May 31, 2017
Merck Serono Co., Ltd.,	January 1, 2016	December 31, 2016
Nippon Chemiphar Co., Ltd.,	January 1, 2016	December 31, 2016
TOYAMA CHEMICAL CO., LTD.,	April 1, 2016	March 31, 2017
Bayer Yakuhin, Ltd.,	January 1, 2016	December 31, 2016
UCB Japan Co., Ltd.,	January 1, 2016	December 31, 2016
AYUMI Pharmaceutical Corporation,	April 1, 2016	March 31, 2017
CELGENE CORPORATION,	January 1, 2016	December 31, 2016
Senju Pharmaceutical Co., Ltd.	April 1, 2016	March 31, 2017
Bristol-Myers Squibb K.K,	April 1, 2016	March 31, 2017
TOA EIYO LTD,	April 1, 2016	March 31, 2017
TSUMURA & CO.,	April 1, 2016	March 31, 2017
Toray Industries, Inc.,	April 1, 2016	March 31, 2017
TERUMO CORPORATION,	April 1, 2016	March 31, 2017
SEIKAGAKU CORPORATION,	April 1, 2016	March 31, 2017
Teikoku Seiyaku Co., Ltd.,	January 1, 2016	December 31, 2016
ASAHI KASEI PHARMA CORPORATION,	April 1, 2016	March 31, 2017
Wakamoto Pharmaceutical Co., Ltd.,	April 1, 2016	March 31, 2017
MOCHIDA PHARMACEUTICAL CO., LTD.,	April 1, 2016	March 31, 2017
Santen Pharmaceutical Co., Ltd.,	April 1, 2016	March 31, 2017

© 2019 Sawano T et al. *JAMA Network Open.*

Pharmaceutical company	Period of the payment data in 2016	
Mylan Seiyaku Ltd.,	January 1, 2016	December 31, 2016
Yakult Honsha Company, Limited.,	April 1, 2016	March 31, 2017
Minophagen Pharmaceutical Co.,	April 1, 2016	March 31, 2017
Taisho Pharmaceutical Co., Ltd.,	April 1, 2016	March 31, 2017
ASKA Pharmaceutical Co., Ltd.	April 1, 2016	March 31, 2017
Meiji Seika Pharma Co., Ltd.,	April 1, 2016	March 31, 2017
NIHON PHARMACEUTICAL CO., LTD.,	April 1, 2016	March 31, 2017
Maruishi Pharmaceutical Co., Ltd.,	April 1, 2016	March 31, 2017
KYORIN Pharmaceutical Co., Ltd.,	April 1, 2016	March 31, 2017
TEIJIN PHARMA LIMITED.,	April 1, 2016	March 31, 2017
ZERIA Pharmaceutical Co., Ltd.,	April 1, 2016	March 31, 2017
SANWA KAGAKU KENKYUSHO CO., LTD.,	April 1, 2016	March 31, 2017
Kaken Pharmaceutical Co., Ltd.,	April 1, 2016	March 31, 2017
Hisamitsu Pharmaceutical Co., Inc.,	March 1, 2016	February 28, 2017
Sanofi K.K.,	January 1, 2016	December 31, 2016
EA Pharma Co., Ltd.,	April 1, 2016	March 31, 2017
Nippon Boehringer Ingelheim Co., Ltd.,	January 1, 2016	December 31, 2016
Torii Pharmaceutical Co., Ltd.,	January 1, 2016	December 31, 2016
AstraZeneca K.K.,	January 1, 2016	December 31, 2016
Sumitomo Dainippon Pharma Co., Ltd.,	April 1, 2016	March 31, 2017
Novartis Pharma K.K.,	January 1, 2016	December 31, 2016
Eli Lilly Japan K.K.,	January 1, 2016	December 31, 2016
ONO PHARMACEUTICAL CO., LTD.,	April 1, 2016	March 31, 2017
Kissei Pharmaceutical Co., Ltd.,	April 1, 2016	March 31, 2017
Eisai Co., Ltd.,	January 1, 2016	December 31, 2016
NIPPON SHINYAKU CO., LTD.,	April 1, 2016	March 31, 2017
AbbVie GK,	January 1, 2016	December 31, 2016
Mitsubishi Tanabe Pharma Corporation,	April 1, 2016	March 31, 2017
Research Institute for Microbial Diseases,	April 1, 2016	March 31, 2017
MSD K.K.,	January 1, 2016	December 31, 2016
Janssen Pharmaceutical K.K.,	January 1, 2016	December 31, 2016
Kyowa Hakko Kirin Company, Limited,	January 1, 2016	December 31, 2016
Takeda Pharmaceutical Company Limited.,	April 1, 2016	March 31, 2017

© 2019 Sawano T et al. *JAMA Network Open.*

Pharmaceutical company	Period of the payment data in 2016	
TAIHO PHARMACEUTICAL CO., LTD.,	January 1, 2016	December 31, 2016
Otsuka Pharmaceutical Co., Ltd.,	January 1, 2016	December 31, 2016
DAIICHI SANKYO COMPANY, LIMITED.,	April 1, 2016	March 31, 2017
GlaxoSmithKline K.K.,	January 1, 2016	December 31, 2016
Shionogi & Co,. Ltd.,	April 1, 2016	March 31, 2017
Chugai Pharmaceutical Co., Ltd.,	January 1, 2016	December 31, 2016
Novo Nordisk Pharma Ltd.,	January 1, 2016	December 31, 2016
Astellas Pharma Inc.,	April 1, 2016	March 31, 2017
Pfizer Japan Inc.,	December 1, 2015	November 30, 2016
Otsuka Holdings Co.,Ltd.,	January 1, 2016	December 31, 2016
Otsuka Pharmaceutical Factory, Inc.	January 1, 2016	December 31, 2016
EN Otsuka Pharmaceutical Co., Ltd.	January 1, 2016	December 31, 2016
Taisho Toyama Pharmaceutical Co., Ltd.	April 1, 2016	March 31, 2017
Biofermin Seiyaku Co., Ltd	April 1, 2016	March 31, 2017
BEE BRAND MEDICO DENTAL CO., LTD.	April 1, 2016	March 31, 2017

© 2019 Sawano T et al. *JAMA Network Open.*
